# A Biomechanical Model of the Scapulothoracic Joint to Accurately Capture Scapular Kinematics during Shoulder Movements

**DOI:** 10.1371/journal.pone.0141028

**Published:** 2016-01-06

**Authors:** Ajay Seth, Ricardo Matias, António P. Veloso, Scott L. Delp

**Affiliations:** 1 Department of Bioengineering, Stanford University, Stanford, California, United States of America; 2 Universidade de Lisboa, Faculdade de Motricidade Humana, LBMF, Lisbon, Portugal; 3 School of Healthcare, Setúbal Polytechnic Institute, Setúbal, Portugal; 4 Department of Mechanical Engineering, Stanford University, Stanford, California, United States of America; University of Manchester, UNITED KINGDOM

## Abstract

The complexity of shoulder mechanics combined with the movement of skin relative to the scapula makes it difficult to measure shoulder kinematics with sufficient accuracy to distinguish between symptomatic and asymptomatic individuals. Multibody skeletal models can improve motion capture accuracy by reducing the space of possible joint movements, and models are used widely to improve measurement of lower limb kinematics. In this study, we developed a rigid-body model of a scapulothoracic joint to describe the kinematics of the scapula relative to the thorax. This model describes scapular kinematics with four degrees of freedom: 1) elevation and 2) abduction of the scapula on an ellipsoidal thoracic surface, 3) upward rotation of the scapula normal to the thoracic surface, and 4) internal rotation of the scapula to lift the medial border of the scapula off the surface of the thorax. The surface dimensions and joint axes can be customized to match an individual’s anthropometry. We compared the model to “gold standard” bone-pin kinematics collected during three shoulder tasks and found modeled scapular kinematics to be accurate to within 2mm root-mean-squared error for individual bone-pin markers across all markers and movement tasks. As an additional test, we added random and systematic noise to the bone-pin marker data and found that the model reduced kinematic variability due to noise by 65% compared to Euler angles computed without the model. Our scapulothoracic joint model can be used for inverse and forward dynamics analyses and to compute joint reaction loads. The computational performance of the scapulothoracic joint model is well suited for real-time applications; it is freely available for use with OpenSim 3.2, and is customizable and usable with other OpenSim models.

## Introduction

The human shoulder is a complex mechanism that enables a wide range of motion and provides the maneuverability and structural support necessary to perform day-to-day activities and athletic feats [1]. While the motion of the scapula is often used as a diagnostic indicator of shoulder pathology [2], scapular kinematics are difficult to characterize [3] due to the scapula’s movement below muscle, fat, and skin. Recent bone-pin measurements indicate that differences between symptomatic and asymptomatic individuals exist and that, although differences are small, they are significantly larger than measurement errors [4]. De Baets et al. 2013 [5] showed that kinematics could be reliably measured from surface markers in stroke patients (and control subjects) for movements with a large range of motion and for a subset of scapula degrees-of-freedom. Biomechanical models are used to improve the accuracy of joint kinematic measurements during walking (e.g. [6–8]). We reasoned, therefore, that a model of the shoulder may make it possible to estimate the motion of the scapula with sufficient accuracy to distinguish between symptomatic and asymptomatic individuals using surface markers. This can be achieved if a shoulder model can: attenuate the effects of noisy surface marker data, permit the range of possible kinematics of the scapula, and detect changes in scapular kinematics in response to forces and differences in morphology. A shoulder model would be valuable if it enables both kinematic and dynamic analyses, and could perform these analyses efficiently to provide clinicians and patients with useful feedback at the time of an experiment in which the motion of surface markers is measured.

We have developed a biomechanical model of the scapulothoracic joint capable of fast and accurate kinematic and dynamic analyses. The scapulothoracic joint model is based on an internal coordinate joint formulation [9] that captures the biomechanically permissible kinematics and enforces the motion of the scapula without kinematic surface constraints. The goals of this study are to: 1) evaluate the accuracy of scapular kinematics from the model of the scapulothoracic joint against “gold standard” bone-pin data measured during shoulder flexion, abduction, and internal rotation tasks; 2) test whether the model can reduce kinematic error due to systematic and random noise in marker trajectories; and 3) assess the computational performance of the model for inverse kinematics, inverse dynamics, and forward dynamics simulations.

### Scapula Mechanics

Several shoulder models have characterized the mechanics of the scapula in the study of upper-extremity function [10–13], as summarized in Table 1. We briefly review the current models of scapula mechanics for the purpose of musculoskeletal simulation of the upper extremity.

**Table 1:** Summary of models with scapula mechanics. Published models are listed with their modeling approach along with their suitability for real-time analyses and their accessibility for modification and re-use by other researchers and clinicians.

Model	Kinematics	Constraints	Dynamics	Real-time	Accessible
**van der Helm 1994** [10]	gliding plane	3	inverse	no	no
**Garner & Pandy 1999** [11]	gliding plane	3	inverse	no	no
**Maurel & Thalman 2000** [14]	gliding point	2	inverse	no	no
**Holzbaur et al. 2005** [12]	regression	5	no	no	yes
**Dickerson et al. 2007** [15]	regression	5	inverse	no	no
**Blana et al. 2008** [13]	gliding plane	3	yes	no	no
**Chadwick et al. 2014** [16]	no	0	forward	yes	no
**Saul et al. 2015** [17]	regression	5	yes	no	yes
**current model**	gliding plane	0	yes	yes	yes

#### Scapular kinematics

The International Society of Biomechanics (ISB) recommended standard for describing the motion of the upper-extremity including the scapula [18] has been adopted by several models [10–12, 16, 17]. This standard defines the kinematics of the scapula in terms of a body-fixed Euler rotation sequence for a frame fixed to the acromion (*Anglus Acromialis*) of the scapula relative to a frame fixed to the clavicle, assuming a ball joint between these segments. Although ISB-recommended angles have been the standard description for scapular kinematics for nearly a decade, a consistent set of “normal” scapular kinematics for a standardized set of tasks has yet to be established. In contrast, clinical gait analysis routinely utilizes deviation from normative data [19] as a tool to assess the severity of gait pathology and to evaluate treatment outcomes. Normative data for shoulder motions would provide a stronger scientific basis for evaluation of shoulder function.

The rotations (Euler angles) and translations of the scapula are coupled [20]. As the scapula moves on the thoracic surface, it rotates to maintain congruity with the thoracic surface while under load. Several models kinematically constrain the scapula to glide on the thorax surface (e.g., [10, 11, 14]), in agreement with the motion described by Dvir and Berme [21] from X-ray photographs. This gliding plane motion has been represented by one or two fixed scapula points constrained to an ellipsoid surface [10], leading to 5 or 4 degrees of freedom (dof) of the scapula, respectively. These models enable the full motion of the scapula, but have shown to result in unrealistic scapula motion (e.g., [14]) and are computationally costly due to the simultaneous solution of constraint forces. Recently, Bolsterlee et al. [22] demonstrated that the constrained kinematic optimization solution implemented in the Delft Shoulder Elbow Model [10] can lead to nonphysical positions and may cause inaccuracies in model predictions when the scapulothoracic constraints must be satisfied, especially at higher humeral elevation angles. Another approach specifies the orientation of the scapula and clavicle bones by means of regression equations as a function of the humeral angles [23] for example [12, 13, 24, 25] which is often referred to as the “scapulohumeral” or shoulder rhythm. These models can emulate healthy flexion and abduction movements, but fail to represent independent scapula and humerus motions like shrugging, and by definition cannot discriminate between normal and pathological scapular kinematics. Therefore, neither the rigidly constrained scapula nor the coupled-regression solutions have adequately modeled scapular kinematics.

#### Scapula Dynamics

The motion of the scapula is influenced by muscle forces and joint reaction forces, which arise from the thoracic surface as well as the acromioclavicular and glenohumeral joints. Muscle forces are dependent on scapular kinematics, which determine the length, velocity and effective moment arms of the large muscles that attach to the scapula. Joint reaction forces, especially between the scapula and thorax, are primarily a consequence of muscle loads. The more the structure of the scapulothoracic contact restricts the motion of the scapula in the direction of muscle forces, the greater the reaction forces will be.

To solve for the acceleration of a constrained multibody system, both applied forces and constraint reaction forces must be accounted for and the mass matrix must be inverted. Here a fundamental numerical problem arises since applied forces are large and the mass of segments are small, thus the solution for system accelerations approaches a singularity. This problem has been widely ignored. Several models of the upper extremity (e.g., [12]) were not built to solve the dynamics problem, while others struggle with poor performance [10] or eliminate the complex motion of the scapula [26]. Chadwick and colleagues recently achieved real-time performance during forward dynamics by implicit numerical integration methods that address the stiff dynamics due to scapulothoracic contact forces [16]; however, the model cannot be used for purely kinematics analyses and the accuracy of simulated motion has not been verified against experimental measures.

The inability to accurately and reliably describe the kinematics of the scapula and/or to enforce physiological scapula motion during dynamic simulations (Table 1) necessitates a new approach to modeling and simulating scapula mechanics.

## Model and Methods

We developed a model of the scapulothoracic joint using a new formulation of the internal coordinate joint, called a *mobilizer* [9]. We then evaluated: 1) the model’s accuracy by comparing computed shoulder kinematics with motions measured during bone-pin experiments; 2) its robustness by assessing its ability to reject simulated noise; 3) its computational performance by timing simulations in comparison to real time. Details are presented below.

### A model of the scapulothoracic joint

#### Scapulothoracic Joint Definition

**Fig 1:**
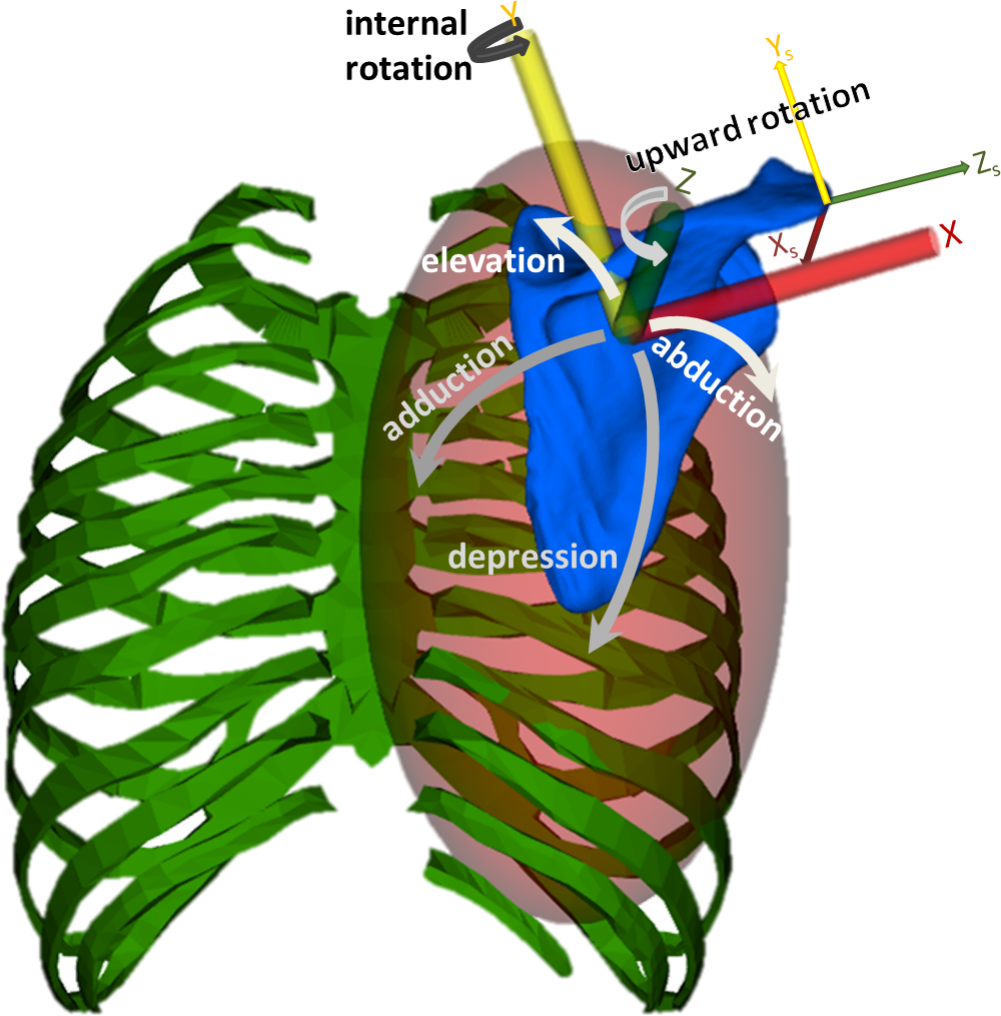
The four modeled degrees of freedom of the scapulothoracic joint. The joint reference frame on the scapula (axes X,Y,Z) is used to locate the scapula with respect to the thorax. The joint reference frame on the scapula is computed according to the ISB recommendations [18] (shown as X_S_, Y_S_, Z_S_), however our joint origin is located at the centroid of the anatomical markers used to define the joint frame instead of the *Angulus Acromialis* and its axes are rotated -90° about Y (to enable positive upward rotation about Z). The joint frame on the thorax defines the center of the scapulothoracic surface modeled as an ellipsoid (red shaded surface). Abduction (adduction) followed by elevation (depression) locate the joint frame origin of the scapula (blue) on the ellipsoid fixed to the parent thorax body (green). The scapula rotates upward (downward) about the normal to the surface (scapula Z-axis). Internal rotation or “winging” is a positive rotation about the Y-axis of the joint frame in the scapular plane, which remains tangent to the thoracic surface.

We characterized the scapulothoracic joint by the translation and rotation of the scapula on the surface of the thorax modeled as an ellipsoid [9, 10]. The scapulothoracic joint defines the kinematics of a joint frame on the scapula with respect to a joint frame on the thorax body. We parameterized scapulothoracic motion (Fig 1) by four position coordinates: 1) *abduction-adduction* [10], 2) *elevation-depression* [10], 3) *upward rotation* [27], and 4) *internal rotation* or “winging” [10, 27, 28]. The first two coordinates, abduction and elevation, locate the origin of the joint reference frame on the thoracic (ellipsoid) surface and are analogous to how longitude and latitude are used to locate a point on the Earth’s surface. The third coordinate, upward rotation, rotates the scapula about the normal to the thoracic surface at the origin of the joint frame. The fourth coordinate, winging, rotates the scapula about a longitudinal axis in the plane of the scapula (which remains tangent to the thoracic surface) and enables the medial border and the *Angulus Inferior* of the scapula to lift off the thoracic surface. The origin of the joint frame on the scapula is specified as the centroid of the *Angulus Acromialis* (AA), *Trigonum Spinae* (TS) and *Angulus Inferior* (AI) markers, which are the anatomical landmarks recommended by the ISB [18]. The orientation of the joint frame on the scapula is also computed according to the ISB recommendations; however, the scapulothoracic joint frame is rotated -90° about the scapular Y-axis to define upward rotation as a positive rotation about the scapulothoracic joint frame’s Z-axis (Fig 1).

#### The Mobilizer Formulation

Internally, the scapulothoracic joint is composed of two mobilizers: an ellipsoid mobilizer [9] and a pin mobilizer [29]. A mobilizer can be thought of as the mathematical dual of a constraint—while a constraint removes dofs from a model, a mobilizer grants dofs to a body, which we term the body’s “mobilities”. Unlike typical engineering joints, which can be reconstituted by a series of ideal 1-dof joints [14], basically pins and sliders, the mobilizer enables smooth and continuous spatial motion between two bodies parameterized by 1–6 internal coordinate speeds or mobilities. This formulation is utilized by Simbody [29], an open source multibody solver available from Simtk.org, and serves as the computational foundation of OpenSim [30, 31].

In Simbody, the kinematics of a body, B, with respect to its parent, P, are fully described by the following four mobilizer equations:

$${}^{P}X^{B}\equiv \left[\;{}^{P}R^{B}(q)\;{}^{P}p^{B}(q)\;\right]$$ (1)

$${}^{P}V^{B}\equiv \left\{\begin{array}{c} {}^{P}\omega^{B}(q,u)\\ {}^{P}v^{B}(q,u) \end{array}\right\}={}^{P}H^{B}(q)\cdot u$$ (2)

$${}^{P}A^{B}\equiv {}^{P}{\dot{V}}^{B}={}^{P}H^{B}\dot{u}+{}^{P}{\dot{H}}^{B}u$$ (3)

$$\dot{q}=N(q)u$$ (4)

Eq (1) describes the position transform, ^*P*^**X**^*B*^, composed of the rotation matrix, **R**, and translation vector, *p*, of a mobilizer frame, B, fixed in the body (B_o_ frame) with respect to a parent mobilizer frame, P, fixed in the parent body (P_o_) (e.g., scapula body and thorax as parent in Fig 1). The spatial velocity, ^*P*^*V*^*B*^ (composed of angular velocity vector *ω* and linear velocity vector *v*) in Eq (2), and acceleration, ^*P*^*A*^*B*^ in Eq (3), of B with respect to P, are specified by the mobilizer matrix, **H**, and its time derivative, $\dot{H}$. The evolution of the internal coordinates, *q*, is governed by the differential relationship Eq (4) with the mobilities, *u*, according to the kinematic coupling matrix, **N**. While constant **H** matrix captures the kinematics of most typical engineering joints (e.g., pin or ball-and-socket), if we do not assume **H**(*q*) to be constant, **H**(*q*) can represent curvilinear paths in the 6-dimensional spatial kinematics basis of a body with respect to its parent. For instance, **H**(*q*) can represent movement about a helix or slider path that is curved in space (curvy slot), or other coupled motions, which are typical of biological joints [29].

The ellipsoid mobilizer of the scapulothoracic joint enables the abduction (adduction), elevation (depression) and upward (downward) rotation of the scapula with respect to the thorax. The scapulothoracic ellipsoid mobilizer has the same 3 mobilities, *u*, as a ball-and-socket, which are the components of the angular velocity of the child (scapula) joint frame with respect to the parent joint frame on the thorax (center of the ellipsoid). However, rather than grant 3 additional mobilities for spatial translations, which would then have to be constrained, the translations are coupled to the orientation of the scapula:

$${}^{T}X^{S}=\left[\;{}^{T}R^{S}(q)\;p(q)\;\right],$$ (5)

where *q* is the scapula-fixed Euler angle sequence relating to abduction (*θ*_1_), elevation (*θ*_2_) and upward rotation (*θ*_3_, about the ellipsoid normal). The vector *p*(*q*) describes the translation of the scapula’s joint frame, *S*, on the surface of an ellipsoid, fixed in the thorax joint frame, *T*:

$$p(q)=\left\{\begin{array}{c} h\;n_{1}\\ w\;n_{2}\\ d\;n_{3} \end{array}\right\}=\left\{\begin{array}{c} h\;\text{sin}(\theta_{2})\\ w\;(-\text{sin}(\theta_{1})\text{cos}(\theta_{2})\\ d\;\text{cos}(\theta_{1})\;\text{cos}(\theta_{2}) \end{array})\right\}$$ (6)

where *n = n(q)* is the surface normal expressed in the thorax, and *h*, *w*, *d* are the ellipsoid radii along the axes of *T* that correspond to height, width and depth, respectively. The angular velocity remains the same function of *u* as for a ball-and-socket joint, so **N** is

$$N(q)=\left[\begin{array}{ccc} \text{cos}\;\theta_{3}\;/\;\text{cos}\;\theta_{2} & -\text{sin}\;\theta_{3}\;/\;\text{cos}\;\theta_{2} & 0\\ \text{sin}\;\theta_{3} & \text{cos}\;\theta_{3} & 0\\ -\text{sin}\;\theta_{2}\;\text{cos}\;\theta_{3}\;/\;\text{cos}\;\theta_{2} & \text{sin}\;\theta_{2}\;\text{sin}\;\theta_{3}\;/\;\text{cos}\;\theta_{2} & 1 \end{array}\right]$$ (7)

Since there are coupled linear velocities as a consequence of the rotating normal vector, the ellipsoid mobilizer matrix has the following form:

$${}^{T}H^{S}={\left[\begin{array}{cccccc} 1 & 0 & 0 & 0 & -wn_{3} & dn_{2}\\ 0 & 1 & 0 & hn_{3} & 0 & -dn_{1}\\ 0 & 0 & 1 & -hn_{2} & wn_{1} & 0 \end{array}\right]}^{\text{T}}$$ (8)

In this case $\dot{H}$ is nonzero and it describes the angular velocity contributions to the body’s linear acceleration:

$${}^{T}{\dot{H}}^{S}={\left[\begin{array}{cccccc} 0 & 0 & 0 & 0 & -w{\dot{n}}_{3} & d{\dot{n}}_{2}\\ 0 & 0 & 0 & h{\dot{n}}_{3} & 0 & -d{\dot{n}}_{1}\\ 0 & 0 & 0 & -h{\dot{n}}_{2} & w{\dot{n}}_{1} & 0 \end{array}\right]}^{\text{T}}$$ (9)

The mobilizer matrix ^*T*^**H**^*S*^ and its derivative ${}^{T}{\dot{H}}^{S}$ span exactly and map only to the subspace of the permissible-motion manifold of an ellipsoid surface. Representing the same motion with conventional joints would require a free joint (6 dofs) and 3 constraint equations, for a total of 9 differential algebraic equations versus the mobilizer formulation’s 3 ordinary differential equations.

An additional pin mobilizer enables the 4^th^ dof of the scapulothoracic joint, which is internal rotation of the scapula about a winging axis in the plane of the scapula that enables the medial border and the inferior angle (*Angulus inferior*) to raise off the thoracic surface (Fig 1, winging axis is the scapula Y-axis). In the scapulothoracic joint, the pin’s axis is automatically aligned with the “winging” axis specified in the joint frame on the scapula and can be set by the user.

#### Multibody Dynamics

Eqs (1–4) define the kinematics of a body with respect to its parent, thus the spatial kinematics of each body can be computed by recursively traversing the bodies from the ground or root body (e.g., thorax) out to the most distal bodies (e.g., hand). From the most distal bodies we apply forces to determine the spatial acceleration of the body in terms of the generalized coordinates and mobilities (*q*, *u*) and recurse inward towards the ground to form the complete set of system accelerations, $\dot{u}$. This approach describes a recursive Newton-Euler algorithm for solving multibody dynamics equations [32]. When position and velocity kinematics constraint equations are differentiated and expressed in terms of the system accelerations, the complete system dynamics can be written as a system of differential-algebraic equations [33] of this form [29]:

$$M\dot{u}+G^{T}\lambda =f_{applied}-f_{inertial}$$ (10.a)

$$G\dot{u}=b$$ (10.b)

where **M** is the system mass matrix in the mobility space; **G** is the Jacobian matrix of the kinematic constraints; ***b*** contains the coordinate and mobility (speed) terms of the differentiated constraints; *λ* are the constraint Lagrange multipliers corresponding to components of the constraint forces; ***f***_applied_ is the net applied generalized force, and ***f***_inertial_ is the net velocity-based generalized force due to rotating frames. Given system accelerations ($\dot{u}$) we can solve for the required applied forces (inverse dynamics) or use the same set of equations to evaluate accelerations due to applied forces and integrate from initial coordinates and mobilities (*q*_o_, *u*_o_) to compute the trajectory of coordinates and mobilities forward in time (i.e., a forward dynamics simulation).

In the case of the scapulothoracic joint, the motion of the scapula is fully captured by the mobilities of the ellipsoid and pin mobilizers and there are no constraints associated with the joint, however constraints do appear in the general form of the system dynamics (Eq 10) for a shoulder model (e.g. acromioclavicular ball-joint constrains 3 translations). Instead, the joint (mobilizer) reaction loads can be determined by solving the Newton-Euler dynamics per body, for example for the scapula:

$$M_{S}A_{G}^{S}=\sum_{nf}f_{i}+{}^{T}R_{G}{}^{S}+{}^{H}R_{G}^{S}+{}^{C}G_{G}^{S}\lambda_{ac}$$ (11)

where *M*_s_ is the spatial inertia matrix of the scapula and $A_{G}^{S}$ is the spatial acceleration of the scapula in ground (by expressing the acceleration from Eq 3 in ground); *f*_*i*_ is an applied spatial force on the scapula; ^*T*^*R*_*G*_^*S*^ is the unknown thoracic reaction load applied to the scapula expressed in ground; ${}^{H}R_{G}^{S}$ is the known glenohumerual joint reaction load on the scapula expressed in ground; and ${}^{C}G_{G}^{S}\lambda_{ac}$ is the acromial (scapuloclavicular) constraint force (*λ*_*ac*_) on the scapula expressed in ground. The system accelerations ($\dot{u}$) and constraint forces (λ) are determined from Eq 10. Simbody determines the reaction loads as part of computing inter-body spatial forces [34].

The scapulothoracic joint was implemented as an OpenSim Joint and included as part of the OpenSim 3.2 application via a plugin library. Model scaling, inverse kinematics, inverse dynamics and forward dynamics simulations were performed in OpenSim 3.2.

### Evaluation of the Scapulothoracic Joint

#### Shoulder Model

We constructed a shoulder model consisting of the thorax, scapula, clavicle, and humerus to evaluate the accuracy, robustness to noise and computational speed of the scapulothoracic joint. The sternoclavicular joint was modeled by a universal joint enabling protraction-retraction and elevation-depression of the clavicle, since axial rotation cannot be accurately measured and the conoid ligament limits axial rotation. The acromioclavicular joint was modeled as a ball joint. The glenohumeral joint was modeled as a custom gimbal joint using the ISB standard coordinates for angle of the elevation plane, elevation, and internal rotation [18]. Segment dimensions and joint locations were obtained from Holzbaur et al. [12] and the segment inertial properties were obtained from Breteler et al. [35].

#### Reconstruction of Experimental Kinematics

We evaluated the kinematic accuracy of the scapulothoracic joint by comparing thorax, scapula and humerus model marker locations to measured marker data from bone-pin motion-capture experiments [28]. Ludewig et al. measured marker locations with magnetic sensors taped to the thorax on the anterior aspect of the sternum, rigidly attached into the scapula at the scapular spine at the acromial base, into the lateral third of the clavicle and distal to the deltoid insertion on the lateral aspect of the humerus, for three tasks: arm flexion (forward raise), arm abduction (to the side) and internal/external rotation of the upper arm when the humerus is abducted at the glenohumeral joint by 90° with respect to the thorax. The magnetic sensor device (Flock of Birds—Ascension Technology, Burlington, Vermont) had an accuracy of 1.8mm in location and 0.5° in orientation under static conditions at a recording frequency of 120frames/s. Ludewig et al. also used a digitizing stylus to register the anatomical landmarks of the *Angulus inferior* and *Trigonum spinae scapulae* to virtual markers (AI and TS) in the rigidly secured sensor frame, which had a measurement accuracy < 1mm [28]. Combining the orientation error with a mean distance of 10cm for two landmarks of interest with static location and stylus errors, yields a measurement accuracy of 1.85mm for the location of a scapula marker in the ground reference frame.

We scaled upper-extremity segment dimensions to obtain a match between model markers and corresponding experimental marker locations identifying the same bony landmarks. Scaling functionality was provided by the Scale Tool in OpenSim [30, 31].

We customized the location and orientation of the thoracic ellipsoid surface, with respect to the thorax origin and the location and orientation of the joint reference frame with respect to the scapula origin on the acromion according to the ISB recommendations [18], to construct a subject specific model. The origin of the scapulothoracic joint reference frame on the scapula was located at the centroid of the measured anatomical markers (AA, TS, AI) and its axes rotated 90° (see Fig 1). The remaining parameters were determined by minimizing the squared errors between model and subject marker locations from nine poses evenly selected from each task. For each task the ellipsoid orientation and radii were adjusted because marker errors were most sensitive to these parameters and account for rib-cage expansion and spinal bending/twisting differences between the tasks.

Once the upper extremity model was appropriately scaled, we performed inverse kinematics to determine the joint coordinates of the model that minimized the weighted-least-squares error between model marker and corresponding experimental marker locations at each frame of the movement trial [6, 30]. We applied uniform weights for bone-pin marker locations since these markers were all assumed to have the same accuracy. To evaluate the model’s kinematic accuracy, we calculated the root-mean-squared (RMS) errors for each marker across each trial.

#### Inverse Kinematics from Noisy Marker Data

We evaluated the robustness of kinematics determined from the scapulothoracic joint model by introducing errors in the experimental bone-pin marker locations to simulate the effects of using surface markers attached to the skin. The purpose of the noise model was to test the effect of random error and bias in the markers movements relative to the bone, such as stretching, translation and rotational offsets, as well as warping. The noise model combines a systematic bias (as a function of the movement) and a random component (for the offset direction and white noise), and we tested a range of error magnitudes. We specified the mean and standard deviation of the noise distribution for each marker at each instant in the movement. We used skin marker errors (location on skin surface to corresponding fixed bony landmark location) measured by MRI [36] to specify the mean of the added noise. The mean of the noise over the task trial was modeled as a Gaussian function over time where the timing of the peak coincided with the time of maximum arm elevation (or rotation), which was the posture in which the maximum skin error was measured by Matsui and colleagues [36]. The Gaussian function gradually scaled the mean skin noise (i.e. the bias) to zero in the neutral static start and end postures. Mean skin noise levels were varied by 1mm increments from 1mm to the mean measured maximum skin surface error (41mm, [36]) at maximum elevation to represent varying amounts of skin movement artifact. We used the standard deviations of the skin surface error from experiments [36] in the simulated noise distribution to reflect random errors, which were included at all noise levels. Noise was added as offsets in marker locations at every instant in time with the direction of the offset also selected at random, sampled from a uniform distribution, for each marker and held constant for each trial. We synthesized 100 trials of “noisy” marker data for each noise level to generate a dataset containing a host of skin movement artifacts dictated by the offset directions of the markers. For example, markers whose noise directions were similar represented a shifting bias (translational offset); opposite directions represented stretching or compressing (depending on whether the directions were away from or towards one another), while other directions reflect rotational shifts and combinations of artifacts.

We computed Euler angles from the noisy marker data according to the ISB standard [18] after lowpass filtering the marker data at 5Hz to duplicate clinical estimates of scapular kinematics with respect to the thorax. We also determined Euler angles from the model’s markers affixed to the scapula after performing inverse kinematics from noisy marker data for each noise level (41) and each activity (3) for a total of 12,300 trials. We compared the mean error and standard deviations of Euler angles with and without the model, as well as those of the scapulothoracic joint coordinates, from trials with noise against those obtained from bone-pin data.

#### Computing Scapulothoracic Forces that Generate Shoulder Movements

We next performed an inverse dynamics analysis to calculate the generalized forces necessary to produce the reconstructed shoulder motions (i.e., the joint (generalized) coordinates, velocities, and accelerations). Coordinates were determined from inverse kinematics of bone-pin measurements. A third order lowpass Butterworth digital filter with a 2Hz cut-off was used to filter the coordinate data and a generalized cross-validated quintic spline [37] was used to interpolate the filtered data. Spline functions for each coordinate were differentiated to obtain velocities and then again to obtain coordinate accelerations as inputs to inverse dynamics.

#### Forward Dynamics Simulation of Shoulder Motion

The multibody dynamic equations of motion were used to determine the acceleration of the joint coordinates in response to applied forces. The velocities and accelerations were integrated forward in time to yield the position and velocity of the coordinates to generate a simulation of passive arm swing. Passive force elements prevented the scapula and humerus from reaching non-physiological positions. We initialized the simulation to be at rest at the maximum humerus elevation angles calculated from the bone-pin trial of each task, then allowed the arm to swing passively for a duration of 2s. The total system energy was computed and monitored during each simulation above. We used a variable step Runge-Kutta-Merson integration algorithm [38] with a relative accuracy of 1e-4 for all forward dynamics simulations, with constraints maintained to 0.01mm.

## Results

### Scapular kinematics from experimental bone-pin measurements

After scaling the shoulder model, including the dimensions of the scapulothoracic joint, the inverse kinematics of the joint were in excellent agreement with bone-pin measurements. On average, the model was accurate to within the measurement error (1.9mm model vs. 1.85mm measurement accuracy, worst case 2.4mm) of the bone-pin markers from the experimental data collection procedure (Table 2).

**Table 2:** Marker errors between model reconstruction and bone-pin experiments (RMS in mm) from three shoulder activities. The mean error across all markers and the worst marker for a task are also provided. Scapula markers include *Angulus Acromialis* (AA), *Angulus Inferior* (AI) and *Trigonum Spinae* (TS). Thorax markers include *Incisura Jugularis* (IJ), the spinous process of the seventh cervical (C7) and eighth thoracic (T8) vertebra. Humerus markers include the glenohumeral joint center (GH) and the lateral (EL) and medial (EM) epicondyles. Marker definitions from [18].

Activity	Scapula Markers			Thorax Markers			Humerus Markers			Mean	Worst
	AA	AI	TS	IJ	C7	T8	EL	GH	EM		
**Flexion**	1.2	2.4	2.0	2.3	1.7	2.1	1.9	1.9	1.7	1.9	AI
**Abduction**	1.9	1.7	1.4	1.6	1.7	1.5	1.9	2.4	1.8	1.8	GH
**Int./Ext.@90°abd**	0.6	1.6	1.8	2.3	1.2	0.9	0.4	1.0	1.3	1.1	IJ

**Fig 2:**
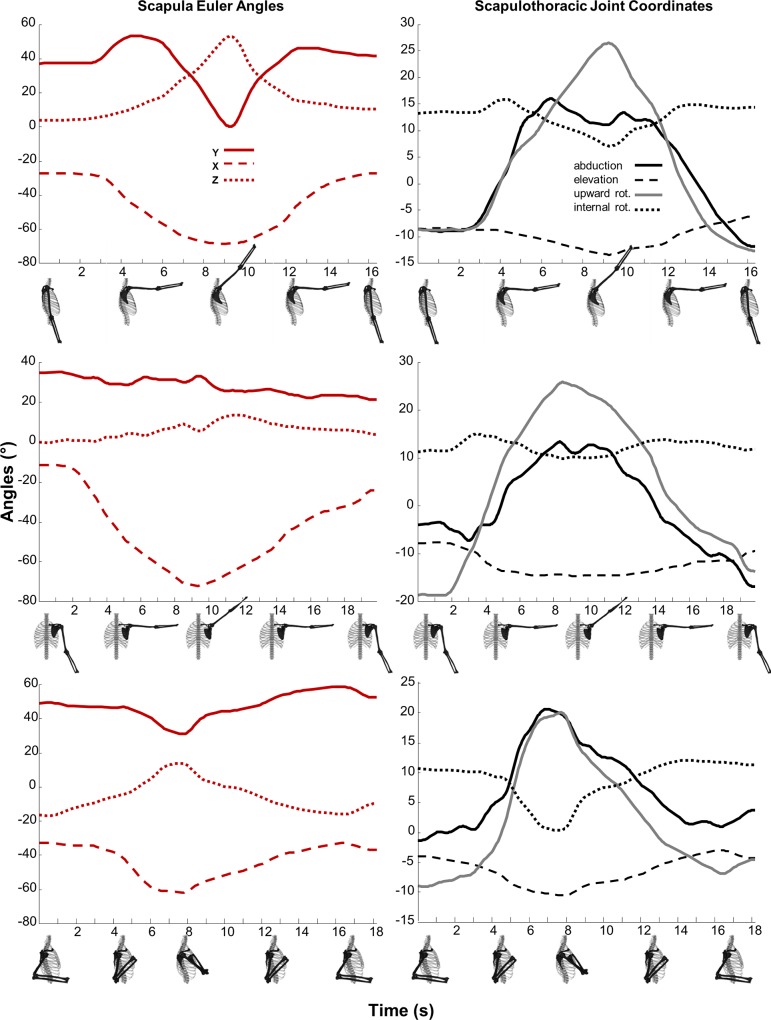
Scapula and Scapulothoracic joint kinematics during shoulder flexion, abduction, and rotation tasks. Scapular kinematics described by the relative rotation of the scapula with respect to the thorax expressed as a body-fixed Y-X-Z Euler angle sequence according to the ISB standard (left panel: Y-internal rotation, solid; X-downward rotation, dashed; Z-posterior-tilting, dotted) and the scapulothoracic joint coordinates (right panel) with abduction (black solid), elevation (dashed), upward rotation (gray solid), and internal rotation or winging (dotted) reconstructed motion from measured bone-pin marker locations during shoulder tasks of: flexion, abduction, and rotation at 90° of humerus abduction.

The scapular kinematics resulting from the scapulothoracic joint in ISB standard rotation sequence (Fig 2, left panels) are similar to the kinematics reported by McClure et al. [27]. The internal coordinate trajectories (Fig 2, right panels) indicate that the motion of the scapula during the flexion task is dominated by the upward rotation of the scapula and reaches a peak of 27° at maximum flexion. Upward rotation combined with scapular abduction represented the two principle modes of scapula motion during the flexion task. In fact, the scapula experienced a slight depression (also observed by [21]) and small fluctuation in winging in the healthy subject. The abduction task was dominated by scapula upward rotation and abduction; however, the degree of abduction was reduced and the initial posture was more downward rotated (Fig 2, middle row right panel). The task of shoulder rotation at 90° of humerus abduction, as expected, started at a pose that was identifiably similar to the initial motion of the abduction task (at approximately 3s) but as the scapula continues to abduct and rotate upward (similar to the previous tasks) it also externally rotates (dotted line) to hold the glenohumeral joint stationary relative to the thorax.

### Scapular Kinematics from Noisy Marker Measurements

**Fig 3:**
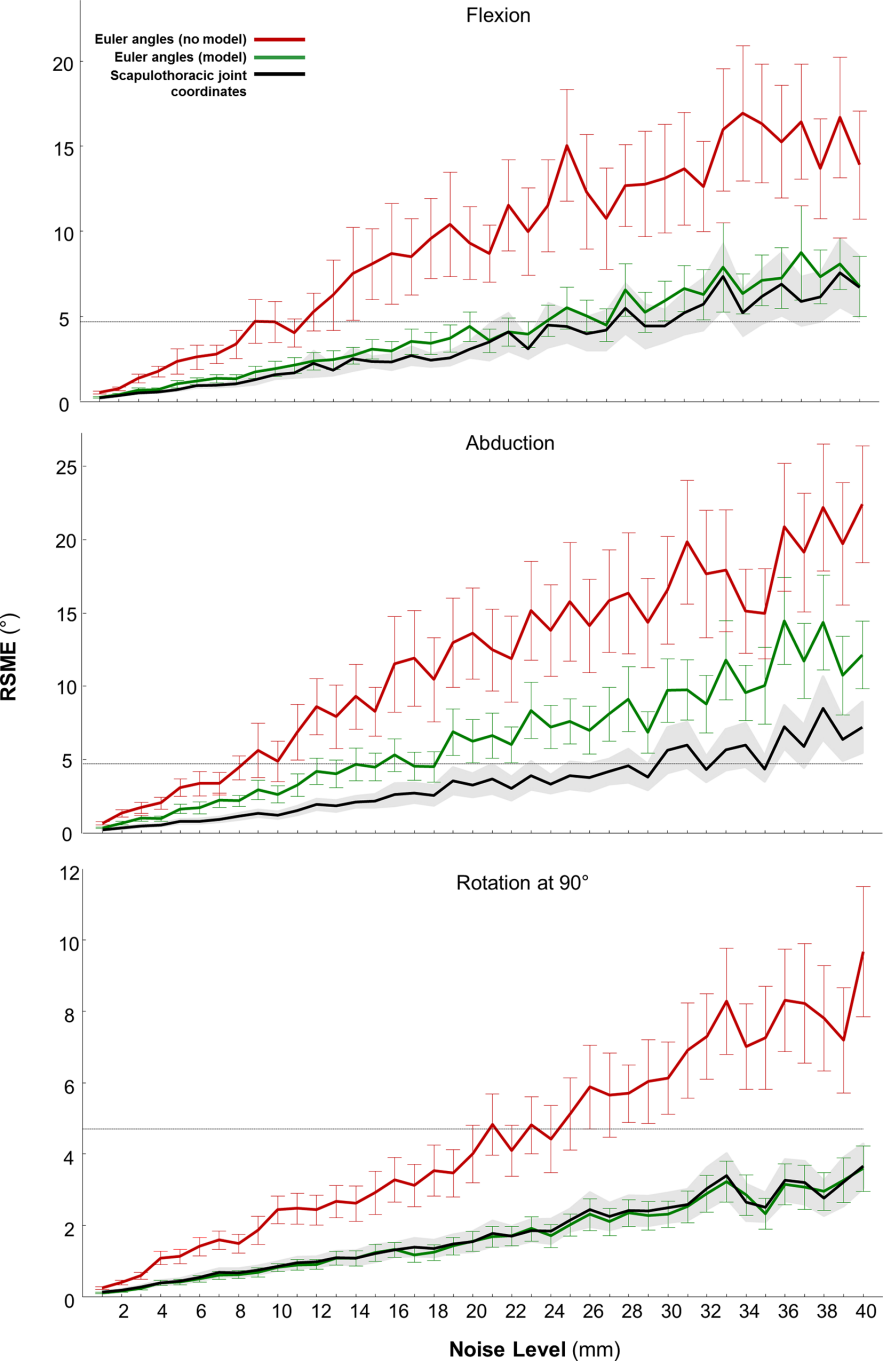
Mean and standard deviation of root-mean-squared errors (RMSE) of scapular kinematics in the presence of noise compared to noise-free kinematics. Scapular kinematics were computed with and without the scapulothoracic joint model during shoulder (humeral) flexion, abduction and rotation tasks. At every noise level, the model (green) and, in particular, the use of the scapulothoracic joint model coordinates (black), reduces RMSE by over 65% compared to direct scapula Euler angle calculations from markers (red). Standard deviations of Euler angles and joint coordinates are indicated by vertical bars and gray shading, respectively. The horizontal dotted line at 4.7° indicates where errors in scapular angles would result in an inability to distinguish the movement between different subjects (Bourne et al. 2011).

Using the scapulothoracic joint model to compute ISB-recommended angles of scapula orientation (Fig 3) reduced the variability in Euler angles due to marker noise by 65% compared to angles computed directly from markers. The reduction in angle errors was consistent over all tasks and levels of noise (Fig 3, red vs. green). We used the intra-subject variability of 4.7° [39] as a worst-case accuracy cut-off for yielding kinematics that are clinically distinguishable from one another. The model-based inverse kinematics could tolerate more than 20mm of marker noise versus only 8mm of noise for the direct marker-based measurement for the same accuracy cut-off.

### Scapulothoracic Kinetics that Generate Shoulder Movements

**Fig 4:**
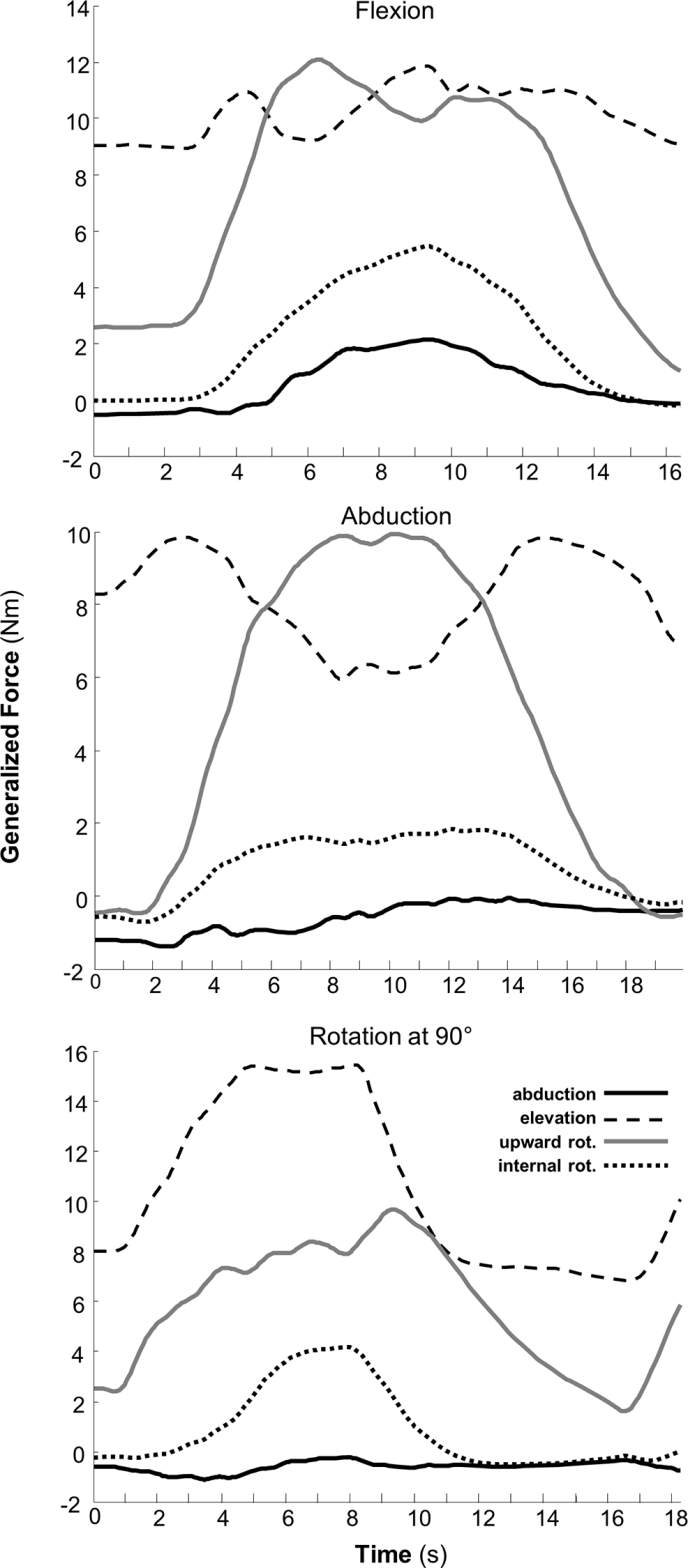
Scapulothoracic generalized coordinate forces (Nm) during shoulder flexion, abduction and rotation at 90° of shoulder abduction tasks. Scapula abduction (bold), elevation (dashed), upward rotation (gray), and internal rotation (dotted) generalized torques computed from an inverse dynamics analysis. A large sustained torque is required to keep the scapula elevated against gravity and requires additional upward rotation torque (gray) to rotate the scapula and lift the humerus during arm elevation tasks.

**Inverse dynamics results** (Fig 4) present the generalized (coordinate) forces necessary to maintain the elevation of the scapula in support of the weight of the upper-extremity and to drive the upward rotation of the scapula to perform the shoulder flexion and abduction tasks. Although scapula abduction is a significant mode of the movement of the scapula (Fig 2), the force required to abduct the scapula on the thoracic surface is small (solid black). There is, however, a large winging torque necessary to support the scapula when the humerus is at maximum flexion.

### Computational Performance of the Scapulothoracic Joint

All kinematic and dynamic analyses of the scapulothoracic joint, including a forward dynamics simulation of passive arm swing, executed faster than real time (Table 3, speed factor > 1). The accuracy of inverse-kinematics was assessed in comparison to experimental bone-pin measurements [28].

**Table 3:** Summary of computational speed and accuracy of scapulothoracic joint mechanics. Performance is presented as a ratio of the real movement duration to the computation period, where a result > 1 represents a factor faster than real time. Computation times evaluated from single threaded calculations on an i7-2820QM 2.4GHz processor.

Analysis	Speed Factor (× real time)	Accuracy	Number of Trials
**Inverse kinematics**	> 1.5×	< 2.4 mm†	12,300
**Inverse dynamics**	> 10×	N/A	12
**Forward dynamics**	> 1.6×	< 1e-4*	6

†Assessed using bone-pins with no added noise.

*The accuracy of forward dynamics simulations is determined by the integration accuracy. This loosely corresponds to the number of significant digits in the resultant state values, which are the generalized coordinates and their speeds. An integrator accuracy of 1e-4 translates to four significant digits in the coordinate values.

## Discussion

We have developed a biomechanical model that captures scapulothoracic kinematics and dynamics. Our model of the scapulothoracic joint produced the kinematics of the scapula from surface markers to within the accuracy of bone-pin measurements (Table 2), which serves to validate the kinematics of the model [40]. Using the scapulothoracic joint to compute scapular kinematics reduced kinematic errors by restricting motion to a physiological space, which attenuated the effects of random and systematic noise in surface marker data (Fig 3). In fact, the scapulothoracic joint produces clinically distinguishable angles when systematic marker noise exceeds 2cm, in contrast to direct angle calculations from markers, which degraded to subclinical accuracy with less than 1cm of noise (Fig 3, black vs. red). Consequently, applying the scapulothoracic joint in model-based reconstruction of shoulder movement from clinical motion-capture data has the potential to distinguish between normal and pathological motion using available surface measurements. The formulation of the scapulothoracic joint enables efficient computational performance with inverse kinematics and forward dynamic simulations running at over 1.5 times faster than real time and inverse dynamics exceeding 10 times faster than real time on a single laptop CPU (Table 3).

The scapulothoracic joint coordinates exhibit less sensitivity to noise than the model-based Euler angle sequence (Fig 3). The ISB-recommended Euler angle sequence of the scapula frame with respect to the thorax describes a scapula free-floating in space as if the scapula were free to rotate independent of its translations. In contrast, the scapulothoracic joint coordinates only span the permissible motion of the scapula where the translations are coupled to its rotations, so that marker noise cannot contribute to motions of the scapula beyond what is permitted by the joint.

The solution of the equations of motion for the upper extremity requires high numerical precision to yield accurate results, since large forces and low body masses can cause the system dynamics to approach a numerical singularity. When constraints are used to enforce the motion of the scapula onto a desired surface (e.g., the thorax in our model) the problem is exacerbated [9]. Although Chadwick et al. (2014) obtained real-time performance by modeling scapulothoracic surface constraints with contact forces, they applied implicit numerical integration methods that required an analytical Jacobian of the whole system dynamics (that is the partial derivative of system accelerations with respect to the system states). This approach does not generalize well to other models (e.g., to the full body) and to the inclusion of models of devices that introduce additional dynamics and states, which are important applications of a biomechanical model.

The mobilizer formulation [9] of the scapulothoracic joint does not share these limitations because even when muscle forces are large, the net force projected on the motion space (i.e., the generalized force) is much smaller and so is the resulting acceleration. In contrast, when limited by constraint forces, the acceleration into the thorax surface can be very sensitive to modest amounts of muscle force, indicative of a poor condition number for the system of equations [41], leading to inaccuracy of computed accelerations. Poor accuracy in system accelerations will result in more numerous integration steps to maintain integration accuracy and degrade computational performance. Because the mechanics of the scapulothoracic joint are defined by mobilizers and not constraints, scapula translation dofs and ellipsoid reaction forces do not appear in the system equations of motion (Eq 10). Consequently, the low mass of the scapula does not require specialized numerical methods for constraint stabilization and does not degrade the condition number of the system as reaction forces become large (e.g., from co-contraction of muscles). While the scapulothoracic reaction forces can be computed efficiently by the mobilizer formulation, the sensitivity of contact forces must be tested against systematic changes to musculoskeletal model parameters, such as joint and muscle attachment locations, e.g. [42], which affect muscle lines of action and their moment-arms.

In addition to greater accuracy and faster computational performance, the scapulothoracic joint yields scapular kinematics that may also be easier to interpret and compare across subjects and populations. The coordinates of the scapulothoracic joint uniquely span the functional kinematics of the scapula while the recommended ISB angles parameterize a (Y-X-Z) gimbal joint at the clavicle. It is difficult to interpret the location and orientation of the scapula from Euler angles of the gimbal joint without visualization of its spatial location. To illustrate this point, consider winging of the scapula (raising of the medial/inferior border off the surface of the thorax), a measure of clinical importance [43, 44]. There is no direct way to assess winging from the ISB recommended angles (Fig 2, left column). In contrast, winging is one of the internal coordinates of the scapulothoracic joint model. The remaining scapulothoracic joint coordinates of abduction and elevation locate the scapula relative to the thorax, similar to longitude and latitude on a globe, and upward rotation, which orients the scapula on the thoracic surface, are straightforward to interpret. Combining the ISB recommendations for defining local coordinate systems and the scapulothoracic joint model could lead to a standard description of scapular kinematics that is more intuitive for clinicians to interpret, compare and discuss while also being less sensitive to measurement noise. The fact that relatively simple arm tasks investigated in this study led to multidimensional scapular movement (Fig 2) highlights the need for scapula modeling even when investigating movements that appear to be simple or planar.

Although the results of the model are promising, there are important limitations to consider. First, the shoulder model employing the scapulothoracic joint was scaled to one healthy subject’s experimental bone-pin data for three shoulder tasks. The small RMS error between the model and experimental markers is, in part, a function of how well the thoracic surface of the scapula is represented by an ellipsoid. For other subjects and particularly those with pathologies, the smooth ellipsoid surface assumption may not be as accurate. Second, skin-surface markers were not measured in conjunction with bone-pin measurements; therefore, we are unable to directly assess the ability of the scapulothoracic joint to attenuate skin movement and other soft-tissue artifacts. The application of systematic and random noise may overestimate the effect of measurement noise on kinematic accuracy, particularly in the direct measurement (no model) case. In practice, the ellipsoid parameters will not be optimized to fit high accuracy data (as performed with the bone-pin measurements) which would also impact reconstruction accuracy. Despite these limitations, for an extensive set of added noise, the model-based reconstruction was at least twice as accurate as without the model, which assures us that under typical circumstances the scapulothoracic joint will not increase the effect of measurement noise. We urge others to test the scapulothoracic joint model for their study population and movements.

The scapulothoracic joint will not alleviate all the difficulties of characterizing scapular kinematics. Particularly, defining the joint frames on the thorax and scapula bodies from clinical data remains challenging. Applying the ISB-recommended frames defined by anatomical landmarks on the thorax and scapula (including stylus measurements) can serve to define the scapulothoracic joint that connects the scapula to the thorax and specify the thorax (ellipsoid) and scapula dimensions. To be effective, we must test the sensitivity of reconstructed kinematics to the joint frame definitions and surface dimensions across multiple subjects. Developing a standard set of poses from which to prescribe reference angles for the humerus (relative to the thorax) and identify the thorax position and orientation in ground (e.g., lab frame), could enable automatic scaling and registration of experimental markers into their model counterparts, for example a rigid acromion marker cluster [5, 45], to provide systematically calibrated models.

We are poised to automate shoulder movement reconstruction from existing marker placement recommendations [18] or to apply inertial measurement units [46] to obtain portable and low cost solutions for obtaining clinical shoulder kinematics. From large populations of data it would be possible to create normative data sets of shoulder kinematics in terms of the scapulothoracic joint coordinates. Furthermore, the same joint model used to characterize kinematics is applicable for analyzing shoulder dynamics, in which case the loads between the thorax and the scapula can be assessed. Finally, the scapulothoracic joint is freely and openly available as source code and as a plugin for OpenSim (at https://simtk.org/home/scapulothoracic) to enable widespread exploration, testing, and adoption.

## References

[1] Veeger HEJ, van der Helm FCT (2007) Shoulder function: the perfect compromise between mobility and stability. J Biomech 40:2119–29 17222853 10.1016/j.jbiomech.2006.10.016

[2] Struyf F, Nijs J, Baeyens J-P, Mottram S, Meeusen R (2011) Scapular positioning and movement in unimpaired shoulders, shoulder impingement syndrome, and glenohumeral instability. Scand J Med Sci Sports 21:352–8 10.1111/j.1600-0838.2010.01274.x 21385219

[3] Srikumaran U, Wells J, Freehill M (2014) Scapular Winging: A Great Masquerader of Shoulder Disorders. J Bone Jt Surg 122:1–13 10.2106/JBJS.M.01031 25031384

[4] Lawrence RL, Braman JP, Laprade RF, Ludewig PM (2014) Comparison of 3-dimensional shoulder complex kinematics in individuals with and without shoulder pain, part 1: sternoclavicular, acromioclavicular, and scapulothoracic joints. J Orthop Sports Phys Ther 44:636–A8 10.2519/jospt.2014.5339 25103135 PMC4684907

[5] De Baets L, Van Deun S, Desloovere K, Jaspers E (2013) Dynamic scapular movement analysis: Is it feasible and reliable in stroke patients during arm elevation? PLoS One 8:e79046 10.1371/journal.pone.0079046 24244414 PMC3823991

[6] Lu TW, O’Connor JJ (1999) Bone position estimation from skin marker co-ordinates using global optimisation with joint constraints. J Biomech 32:129–34 10052917 10.1016/s0021-9290(98)00158-4

[7] Leardini A, Chiari L, Della Croce U, Cappozzo A (2005) Human movement analysis using stereophotogrammetry. Part 3. Soft tissue artifact assessment and compensation. Gait Posture 21:212–25 15639400 10.1016/j.gaitpost.2004.05.002

[8] De Groote F, De Laet T, Jonkers I, De Schutter J (2008) Kalman smoothing improves the estimation of joint kinematics and kinetics in marker-based human gait analysis. J Biomech 41:3390–3398 10.1016/j.jbiomech.2008.09.035 19026414

[9] Seth A, Sherman M, Eastman P, Delp S (2010) Minimal formulation of joint motion for biomechanisms. Nonlinear Dyn 62:291–303 21170173 10.1007/s11071-010-9717-3 PMC3002261

[10] Van der Helm FC (1994) A finite element musculoskeletal model of the shoulder mechanism. J Biomech 27:551–69 8027090 10.1016/0021-9290(94)90065-5

[11] Garner B, Pandy M (1999) A kinematic model of the upper limb based on the visible human project (vhp) image dataset. Comput Methods Biomech Biomed Engin 2:107–124 11264821 10.1080/10255849908907981

[12] Holzbaur KRS, Murray WM, Delp SL (2005) A Model of the Upper Extremity for Simulating Musculoskeletal Surgery and Analyzing Neuromuscular Control. Ann Biomed Eng 33:829–840 16078622 10.1007/s10439-005-3320-7

[13] Blana D, Hincapie J, Chadwick E, Kirsch R (2008) A musculoskeletal model of the upper extremity for use in the development of neuroprosthetic systems. J Biomech 41:1714–1721 10.1016/j.jbiomech.2008.03.001 18420213 PMC2586642

[14] Maurel W, Thalmann D (2000) Human shoulder modeling including scapulo-thoracic constraint and joint sinus cones. Comput Graph 24:203–218

[15] Dickerson CR, Chaffin DB, Hughes RE (2007) A mathematical musculoskeletal shoulder model for proactive ergonomic analysis. Comput Methods Biomech Biomed Engin 10:389–400 17891574 10.1080/10255840701592727

[16] Chadwick EK, Blana D, Kirsch RF, van den Bogert AJ (2014) Real-Time Simulation of Three-Dimensional Shoulder Girdle and Arm Dynamics. IEEE Trans Biomed Eng 61:1947–1956 10.1109/TBME.2014.2309727 24956613 PMC4068297

[17] Saul KR, Hu X, Goehler CM, Vidt ME, Daly M, Velisar A, et al (2015) Benchmarking of dynamic simulation predictions in two software platforms using an upper limb musculoskeletal model. Comput Methods Biomech Biomed Engin 18:1445–1458 10.1080/10255842.2014.916698 24995410 PMC4282829

[18] Wu G, van der Helm FCT, Veeger HEJ, Makhsous M, Van Roy P, Anglin C, et al (2005) ISB recommendation on definitions of joint coordinate systems of various joints for the reporting of human joint motion—Part II: shoulder, elbow, wrist and hand. J Biomech 38:981–992 15844264 10.1016/j.jbiomech.2004.05.042

[19] Schwartz MH, Rozumalski A (2008) The Gait Deviation Index: a new comprehensive index of gait pathology. Gait Posture 28:351–7 10.1016/j.gaitpost.2008.05.001 18565753

[20] Roren A, Lefevre-Colau M-M, Poiraudeau S, Fayad F, Pasqui V, Roby-Brami A (2014) A new description of scapulothoracic motion during arm movements in healthy subjects. Man Ther 20:46–55 10.1016/j.math.2014.06.006 25034959

[21] Dvir Z, Berme N (1978) The shoulder complex in elevation of the arm: a mechanism approach. J. Biomech. 10.1016/0021-9290(78)90047-7 711770

[22] Bolsterlee B, Veeger DHEJ, Chadwick EK (2013) Clinical applications of musculoskeletal modelling for the shoulder and upper limb. Med Biol Eng Comput 51:953–63 10.1007/s11517-013-1099-5 23873010

[23] Groot J De, Brand R (2001) A three-dimensional regression model of the shoulder rhythm. Clin Biomech 16:735–743 10.1016/s0268-0033(01)00065-1 11714550

[24] Karlsson D, Peterson B (1992) Towards a model for force predictions in the human shoulder. J. Biomech. 10.1016/0021-9290(92)90275-6 1733994

[25] Charlton IW, Johnson GR (2006) A model for the prediction of the forces at the glenohumeral joint. Proc Inst Mech Eng Part H J Eng Med 220:801–812 10.1243/09544119JEIM147 17236514

[26] Chadwick EK, Blana D, van den Bogert AJ, Kirsch RF (2009) A real-time, 3-D musculoskeletal model for dynamic simulation of arm movements. IEEE Trans Biomed Eng 56:941–948 10.1109/TBME.2008.2005946 19272926 PMC2971671

[27] McClure PW, Michener LA, Sennett BJ, Karduna AR (2001) Direct 3-dimensional measurement of scapular kinematics during dynamic movements in vivo. J shoulder Elb Surg 10:269–77 10.1067/mse.2001.112954 11408911

[28] Ludewig PM, Phadke V, Braman JP, Hassett DR, Cieminski CJ, LaPrade RF (2009) Motion of the shoulder complex during multiplanar humeral elevation. J Bone Joint Surg Am 91:378–89 10.2106/JBJS.G.01483 19181982 PMC2657311

[29] Sherman MA, Seth A, Delp SL (2011) Simbody: Multibody dynamics for biomedical research. Procedia IUTAM 2:241–261 25866705 10.1016/j.piutam.2011.04.023 PMC4390141

[30] Delp SL, Anderson FC, Arnold AS, Loan P, Habib A, John CT, et al (2007) OpenSim: open-source software to create and analyze dynamic simulations of movement. IEEE Trans Biomed Eng 54:1940–50 18018689 10.1109/TBME.2007.901024

[31] Seth A, Sherman M, Reinbolt JA, Delp SL (2011) OpenSim: A musculoskeletal modeling and simulation framework for in silico investigations and exchange. Procedia IUTAM 2:212–232 25893160 10.1016/j.piutam.2011.04.021 PMC4397580

[32] Featherstone R (1983) The Calculation of Robot Dynamics Using Articulated-Body Inertias. Int J Rob Res 2:13–30

[33] Petzold L (1982) Differential/algebraic equations are not ODE’s. SIAM J Sci Stat Comput 3:367–384

[34] Jain A (2010) Robot and Multibody Dynamics: Analysis and Algorithms. 128

[35] Klein Breteler MD, Spoor CW, Van der Helm FC (1999) Measuring muscle and joint geometry parameters of a shoulder for modeling purposes. J Biomech 32:1191–7 10541069 10.1016/s0021-9290(99)00122-0

[36] Matsui K, Shimada K, Andrew PD (2006) Deviation of skin marker from bone target during movement of the scapula. J Orthop Sci 11:180–4 16568391 10.1007/s00776-005-1000-y

[37] Woltring H (1986) A FORTRAN package for generalized, cross-validatory spline smoothing and differentiation. Adv Eng Softw 8:104–113

[38] Hairer E, Nørsett SP, Wanner G (2008) Solving ordinary differential equations I: nonstiff problems Springer Science & Business

[39] Bourne DA, Choo AM, Regan WD, MacIntyre DL, Oxland TR (2011) The placement of skin surface markers for non-invasive measurement of scapular kinematics affects accuracy and reliability. Ann Biomed Eng 39:777–85 10.1007/s10439-010-0185-1 20967500

[40] Hicks JL, Uchida TK, Seth A, Rajagopal A, Delp S (2014) Is my model good enough? Best practices for verification and validation of musculoskeletal models and simulations of human movement. J Biomech Eng. 10.1115/1.4029304 PMC4321112 25474098

[41] Golub GH, van Van Loan CF (1996) Matrix computations (Johns Hopkins studies in mathematical sciences).

[42] Valente G, Pitto L, Testi D, Seth A, Delp SL, Stagni R, et al (2014) Are Subject-Specific Musculoskeletal Models Robust to the Uncertainties in Parameter Identification? PLoS One 9:e112625 10.1371/journal.pone.0112625 25390896 PMC4229232

[43] Kibler W (1998) The role of the scapula in athletic shoulder function. Am. J. Sports Med. 26:10.1177/03635465980260022801 9548131

[44] Kibler BW, Ludewig PM, McClure PW, Michener L, Bak K, Sciascia AD (2013) Clinical implications of scapular dyskinesis in shoulder injury: the 2013 consensus statement from the “Scapular Summit”. Br J Sports Med 47:877–85 10.1136/bjsports-2013-092425 23580420

[45] Van Andel C, van Hutten K, Eversdijk M, Veeger D, Harlaar J (2009) Recording scapular motion using an acromion marker cluster. Gait Posture 29:123–8 10.1016/j.gaitpost.2008.07.012 18815043

[46] Lebel K, Boissy P, Hamel M, Duval C (2013) Inertial measures of motion for clinical biomechanics: Comparative assessment of accuracy under controlled conditions—Effect of velocity. PLoS One. 10.1371/journal.pone.0079945 PMC3833942 24260324

